# Population-Based Assessment of Determining Predictors for Discharge Disposition in Patients with Bladder Cancer Undergoing Radical Cystectomy

**DOI:** 10.3390/cancers14194613

**Published:** 2022-09-23

**Authors:** Raj A. Kumar, Kian Asanad, Gus Miranda, Jie Cai, Hooman Djaladat, Saum Ghodoussipour, Mihir M. Desai, Inderbir S. Gill, Giovanni E. Cacciamani

**Affiliations:** 1 Catherine & Joseph Aresty Department of Urology, Keck Medicine of USC, University of Southern California, Los Angeles, CA 90033, USA; 2 Bladder and Urothelial Cancer Program, Rutgers Cancer Institute of New Jersey, New Brunswick, NJ 08903, USA

**Keywords:** radical cystectomy, discharge disposition, skilled nursing home, home discharge, marital status, disparities, insurance, population-based cohort study, Premier Healthcare Database

## Abstract

**Simple Summary:**

Our study analyzed 138,151 radical cystectomy patient encounters to determine which patient and facility characteristics are associated with discharge home and discharge to continued rehabilitation facilities. We used multivariate logistic regression to statistically analyze these datapoints while controlling for other variables. We found that older age, single/widowed marital status, female gender, increased Charlson Comorbidity Index, Medicaid, and Medicare insurance and open surgery are associated with Continued Rehabilitation Facility (CRF) discharge.

**Abstract:**

Objective: To assess predictors of discharge disposition—either home or to a CRF—after undergoing RC for bladder cancer in the United States. Methods: In this retrospective, cohort study, patients were divided into two cohorts: those discharged home and those discharged to CRF. We examined patient, surgical, and hospital characteristics. Multivariable logistic regression models were used to control for selected variables. All statistical tests were two-sided. Patients were derived from the Premier Healthcare Database. International classification of disease (ICD)-9 (<2014), ICD-10 (≥2015), and Current Procedural Terminology (CPT) codes were used to identify patient diagnoses and encounters. The population consisted of 138,151 patients who underwent RC for bladder cancer between 1 January 2000 and 31 December 2019. Results: Of 138,151 patients, 24,922 (18.0%) were admitted to CRFs. Multivariate analysis revealed that older age, single/widowed marital status, female gender, increased Charlson Comorbidity Index, Medicaid, and Medicare insurance are associated with CRF discharge. Rural hospital location, self-pay status, increased annual surgeon case, and robotic surgical approach are associated with home discharge. Conclusions: Several specific patient, surgical, and facility characteristics were identified that may significantly impact discharge disposition after RC for bladder cancer.

## 1. Introduction

Radical cystectomy (RC) is the mainstay of treatment for muscle-invasive bladder cancer and refractory non-muscle-invasive bladder cancer [1,2]. Despite significant refinement and standardization over the last several years, RC remains a morbid procedure with significant post-operative complications, readmissions, and mortality [3,4]. For this reason, careful patient selection is critical for successful surgical outcomes. There is little data regarding discharge disposition following RC—either to a Continued Rehabilitation Facility (CRF) (including skilled nursing facility (SNF), acute care rehabilitation, outpatient care rehabilitation, etc.) or directly home. A recent study linked discharge to SNFs with increased rate of readmission [5]. Discharge to SNF among other facilities was associated with nearly 50% higher odds of readmission at both 30 and 90 days following discharge after RC.

The cost of CRF services vary widely by state, length of stay, labor, services required, and vacancy. Per the U.S. Census Bureau, 9.2% of the U.S. population remained uninsured in 2019 [6]. Additionally, many insurance plans deter admission to CRFs, and so patients can incur significant financial burden [7]. Given the likelihood of readmission and financial strain, it is imperative to assess which patients are at risk of admission to CRFs. While several studies have associated measures such as frailty, increased age, and poor exercise tolerance with CRF discharge, there has not been a robust, systematic analysis of predictors of discharge disposition [8,9,10].

We hypothesized that older patients or patients without a system of care (such as having a widowed status) would be more likely to be discharged to an SNF. Additionally, we hypothesized that a self-pay insurance status would indicate CRF discharge. We also felt that the surgical approach may prove to have an impact in terms of recovery and therefore minimize CRF discharge [11].

In this study, we used a population-based approach to evaluate predictors of discharge disposition following RC.

## 2. Materials and Methods

### 2.1. Data Source

We used Premier Healthcare Database (PHD) by Premier Inc. (Charlotte, NC, USA), a large U.S. (n. 1041 contributing hospitals/healthcare systems), hospital-based, service-level, all-payer database that includes inpatient discharge information. Inpatient admissions include over 121 million visits, representing approximately 25% of all annual U.S. admissions [12]. PHD collects a large volume of data that could be identified and analyzed using ICD 9 and 10 codes as has been done in multiple past studies [13].

### 2.2. Study Cohort and Variables

We identified patients diagnosed with bladder cancer (BCa) between 2000 and 2019 and underwent RC. We excluded patients who died during the hospital stay. Patients were allocated into two groups based on discharge disposition after RC: those discharged home (with/without home health services) or to CRFs (Appendix A). We used medical-record-level details of International Classification of Diseases, 9th and 10th (ICD-9 and ICD-10) and diagnosis and Current Procedural Terminology (CPT) codes to identify patients (aged ≥18 years) undergoing RC for BCa, urinary diversion (continent or incontinent) and surgical approach (open or robotic [14,15]) (Appendix B). Data on patient characteristics (age, gender, race, and ethnicity, Charlson comorbidity Index (CCI), marital status, primary health insurance) surgical characteristics (urinary diversion, surgical approach) and facility characteristics (hospital size, annual hospital volume and surgeon volume, hospital location and teaching status, year of surgery, and region (Midwest, Northeast, South, West)) were analyzed.

### 2.3. Statistical Analysis

Annual hospital and surgeon RC volumes were calculated and presented as quintiles. Volumes at or below the 20th percentile were considered “low” and volumes above the 80th percentile were considered “high”. Values between these extremes were considered “intermediate”, as previously described [16]. Continuous and categorical variables were presented as mean and standard deviation, and median and interquartile range (IQR), respectively. A univariate analysis was performed to compare differences in baseline demographics, surgical factors, and facility characteristics between the two cohorts. In the univariate analysis, Kruskal–Wallis, chi-squared (X^2^), and Fisher’s exact tests were used to compare continuous and categorical variables as appropriate. We performed separate multivariable logistic regression models. The multivariable model included variables previously found to be predictors of discharge to CRFs [8,9,10] and significant variables from our preliminary univariate analysis. Nationally representative estimates were achieved using projection weights linked to the Premier Database derived from the 1998 American Hospital Association Annual Survey and validated by the 1998 National Hospital Discharge Survey as previously described [17,18]. A two-tailed test with *p* < 0.05 was considered statistically significant. Data were analyzed using SAS 9.0 software and reported according to guidelines for reporting statistics for clinical research in urology [19].

## 3. Results

### 3.1. Baseline Characteristics

We identified 138,151 patients diagnosed with bladder cancer (BCa) between 2000 and 2019 and underwent RC. Baseline characteristics of the patient population are reported in Table 1. Facility characteristics are reported in Table 2. A weighted total of 138,151 patients was included. A total of 24,922 (18.0%) patients were admitted to SNFs. Median age was 70.0 (IQR:62.0–76.0). Median length of stay was 8.0 days (IQR:7.0–12.0).

**Figure 1:**
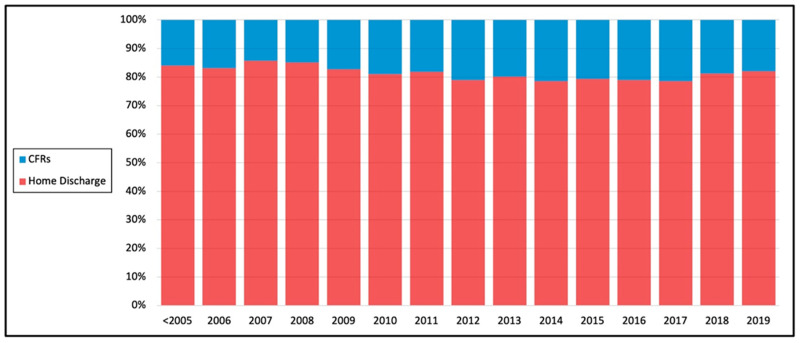
Trends over time of patients discharged home vs. to CFRs in the United States.

Of those discharged home, 94,250 (83.2%) were male and 18,976 (16.8%) female; 65,539 (61.4%) were married and 32,701 (28.9%) single. 22,355 (19.7%) underwent robotic RC and 90,874 (80.3%) underwent open RC. Of those discharged to CRFs, 18,406 (73.9%) were male and 6516 (26.1%) female; 10,962 (44.0%) were married and 11,520 (46.2%) single; 4477 (18.0%) underwent robotic RC and 20,445 (82.0%) underwent open RC. Trends over time showed increasing annual percent of patients discharged to CRF, from 16% before 2005 to 18% in 2019, with a peak of 21.5% in 2017 (Figure 1).

### 3.2. Predictor of Discharge Disposition after RC

**Figure 2:**
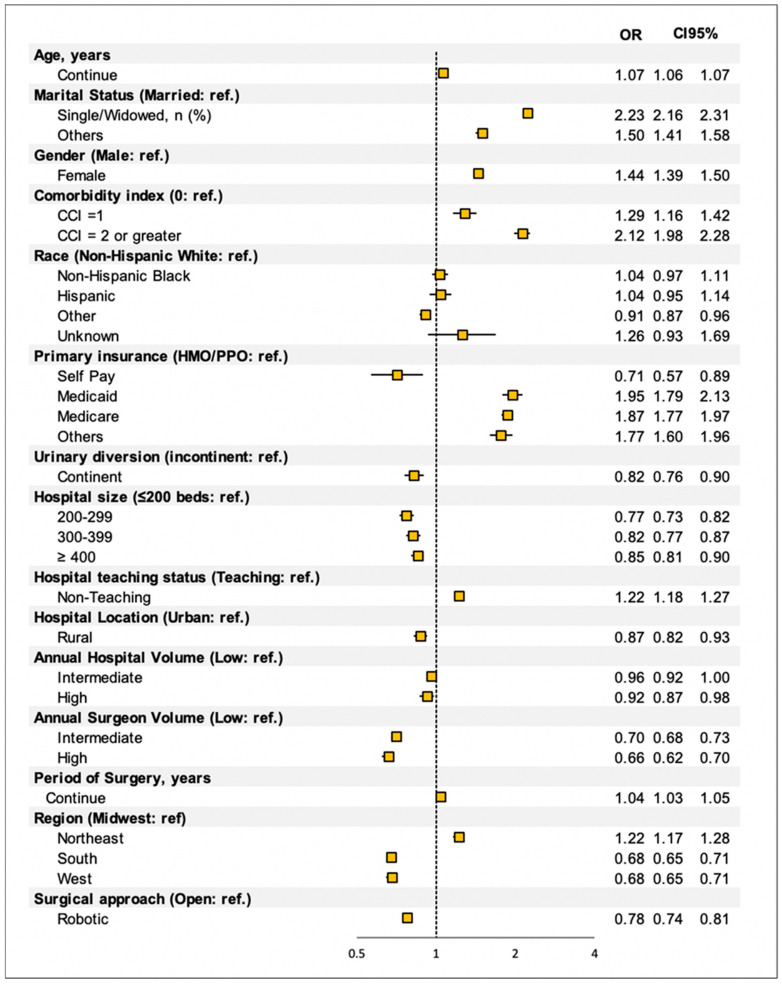
Multivariable Analysis Overall population. OR: Odds Ratio.

Multivariate analysis (Figure 2) revealed that older age, single marital status, female gender, increased CCI score, Medicaid insurance, Medicare insurance, non-teaching hospital status, and northeast geographic location are associated with a significant increase in discharge to CRFs.

Rural hospital location, self-pay status, continent neobladder diversion, 200–299 bed hospital size, increased annual surgeon case volume, and robotic surgical approach are associated with discharge home.

### 3.3. Surgical Volumes-Based Analysis

Multivariate analysis was performed after separating data into high-volume (HV) and non-high-volume (NHV) cohorts (Appendix C). In the HV cohort, increased age, single marital status, female gender, CCI ≥ 2, Medicaid insurance, Medicare insurance, non-teaching hospital status, and northeast geographic location are associated with a statistically significant increase in CRF discharge.

“Other” marital status [rural hospital location, self-pay status, south geographic location, west geographic location, and robotic approach] are associated with discharge home.

In the NHV cohort (Appendix C), increased age, single marital status, “other” marital status, female gender, increased CCI score, “other” race, Medicaid insurance, Medicare insurance, non-teaching hospital status, and northeast geographic location are associated with a statistically significant increase in CRF discharge.

Rural hospital location, self-pay status, 200–299 bed hospital size [south geographic location, west geographic location, and robotic surgical approach] are associated with discharge home.

### 3.4. Geographic Area Analysis

**Figure 3:**
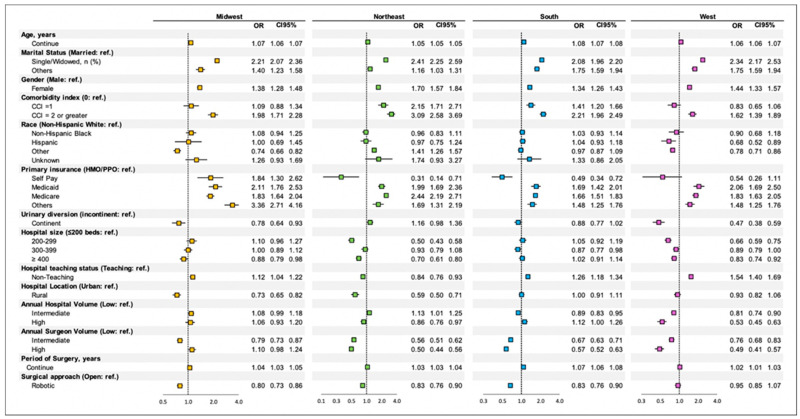
Multivariable Analysis—Geographical Subsets. OR: Odds Ratio.

Multivariate analysis was performed by geographic region: Midwest, Northeast, South, and West. Odds ratios, confidence intervals, and statistical significance are reported in Figure 3. Below we have reported our statistically significant findings.

#### 3.4.1. Midwest

In the Midwest region, increased age, single marital status, female gender, CCI score of 2, “other” insurance, “high” annual surgeon volume, and later year of surgery are associated with a statistically significant increase in CRF discharge.

“Other” race, 400 or more bed hospital size, rural hospital location, “intermediate” annual surgeon volume, and robotic approach are associated with discharge home.

#### 3.4.2. Northeast

In the Northeast region, increased age, single marital status, “other” marital status, female gender, CCI score of 2, Medicaid insurance, Medicare insurance, “intermediate” annual hospital volume, and later year of surgery are associated with a statistically significant increase in CRF discharge.

Self-pay status, 200–299 bed hospital size, 300–399 bed hospital size, non-teaching hospital status, rural hospital location, ‘high” annual hospital volume, “high” annual surgeon volume, “intermediate” annual surgeon volume, and robotic approach are associated with discharge home.

#### 3.4.3. South

In the South region, increased age, single marital status, “other” marital status, female gender, CCI score of 2, Medicaid insurance, Medicare insurance, “other” insurance, non-teaching hospital status, and later year of surgery are associated with a statistically significant increase in CRF discharge.

Self-pay status, 300–399 bed hospital size, “intermediate” annual hospital volume, “high” annual surgeon volume, “intermediate” annual surgeon volume, and robotic surgical approach are associated with discharge home.

#### 3.4.4. West

In the West region, increased age, single marital status, female gender, CCI score of 2, Medicaid insurance, Medicare insurance, and non-teaching hospital status are associated with a statistically significant increase in CRF discharge.

CCI score of 1, continent neobladder diversion, 200–299 bed hospital size, “high” annual hospital volume, and “high” annual surgeon volume are associated with discharge home.

## 4. Discussion

This study evaluates the impact of patient, surgical, and facility factors on discharge disposition of patients with BCa undergoing RC and urinary diversion in the U.S.

Our study has several important findings. First, females, single or widowed patients, and those with higher CCI were significantly more likely to be discharged to a SNF. For these patients, pre-operative counselling should include discussions regarding the increased likelihood of discharge to CRFs. In 2011, Aghazadeh et al. found that older age, poor preoperative exercise tolerance, and longer hospital stay predicted CRF discharge [8]. However, that study included only 445 patients from the same institution (2004–2007). Several studies focused on frailty as an important predictor of discharge to CRFs [9,10]. Though not directly associated with CRF discharge, increased age and female gender were associated with increased frailty. This indirectly supports our findings that age and gender were associated with SNF discharge.

We found patients’ insurance status to be a significant predictor of discharge disposition. Patients who were self-pay were significantly less likely to be discharged to CRFs, while patients with Medicare and/or Medicaid were more likely to be discharged to CRFs. Both Medicaid and Medicare cover SNF stay up to a certain point [20,21]. Medicare Part A covers the entire cost of the first 20 days, and patients will be responsible for a $185.50 co-pay for the next 80 days. Patients will be entirely responsible for any subsequent SNF costs beyond the first 100 days. In 2018, one-fifth of hospitalized Medicare beneficiaries were discharged to SNFs, and Medicare paid a total of $28.5 billion on SNF services [22]. Self-pay patients are responsible for the entire cost and are therefore less likely to desire CRF stay.

Our analysis showed that higher volume surgeons and teaching hospitals were less likely to discharge patients to a CRF. This may be attributable to improved skill, reduced complication rates, use of standardized discharge pathways, and the implementation of standardized protocols including enhanced recovery after surgery (ERAS) protocols [23]. ERAS guidelines for RC were introduced in 2013 [24]. ERAS protocols have been shown to reduce length of stay for patients undergoing radical cystectomy without significant difference in complication rates and readmission rates [25,26].

That said, there is heterogeneity in the application of ERAS protocols between institutions, and even within the same institution [25]. One of the limitations of this study was that PHD did not allow us to control for institutions that have adopted ERAS protocols.

Our study reports a slight increase in discharge to CRF over time. While several novel procedures and technologies have contributed to more optimal surgical outcomes such as minimally invasive surgery and ERAS protocols, we feel that the increase in CRF discharge complements this appropriately. Discharge to CRF provides patients with continued skilled care while also permitting room turnover for more patients. Additionally, with improved surgical outcomes, more patients are eligible for RC. This broader patient pool includes more elderly patients and those with comorbidities that require skilled care even following discharge.

The multivariable analysis has shown that patients undergoing radical cystectomy with a continent urinary diversion are less likely to be discharged to CFR (OR = 0.82, 95% CI 0.76–0.90). This may be explained by targeted patient selection. Continent urinary diversion often warrants a robust selection of candidates that can more strongly tolerate surgical intervention efficiently benefit from a continent urinary diversion. In combination, home discharge, good tolerance of surgery, improved outcomes, and continent diversion may all affect the quality of life in these patients [27,28].

Our study reported that patients who underwent a robotic approach to their surgery were significantly less likely to be discharged to a skilled nursing facility. This may be because robotic RC is associated with decreased length of hospital stay compared to open RC and report fewer complications compared to open surgery [29]. While robotic RC is known to have an increased operative time and cost to the patient, our study shows that this may lead to decreased future costs by avoiding CRF stay. Generally speaking, open RC has been shown to be more cost effective than robotic RC [30]. However, the decreased need for extended CRF stay may impact the cost of robotic RC. Further reports should account for this aspect in the cost-analysis.

Interestingly, we found that both rural hospitals and large, high-volume centers and both associated with home discharge. Though seemingly contradictory, we would reconcile this finding by noting that CRFs in rural areas are smaller and not always available [31]. Larger urban areas are more likely to have available and skilled CRFs, however would more likely utilize this option if patient recovery is slow or if there have been post-surgical complications that warrant skilled nursing staff.

Significant geographic differences were found in CRF discharge across the United States. Patients with a CCI of 2 or greater were nearly twice as likely to be discharged to a CRF in the Northeast compared to the West (OR 3.085 and 1.622, respectively). High annual surgeon volume had more than a twofold greater increase in CRF discharge prediction in the Midwest compared to the West (OR of 1.103 and 0.485, respectively). Additionally, the South has seen the greatest annual increase in CRF discharge over time (OR 1.068 per year), while the West has seen the lowest increase over time (OR 1.016 per year). Insurance status showed the highest degree of variability across geographic region, and protocols based on insurance are highly variable per state and regional regulations.

Finally, we must also recognize the changes to the CRF system over time. Most significantly, in 2006, within 30 days of admission to a nursing facility, nearly 24% of short-stay patients were readmitted to a hospital. Following this, outpatient emergency department use and rehospitalization were added as quality measures for CRFs [32].

Although our findings impact patient preoperative counseling, costs, and outcomes, this must be interpreted within the study limitations. First, the PHD does not provide data on the granularity of cancer staging. Second, we do not have information about institutional adoption and enforcement of ERAS protocols. Third, we do not have data regarding in-hospital complications that could have an impact on discharge disposition as has been previously described [11]. Finally, our study is a retrospective analysis.

There are however several strengths to our study. The large study population allows us to better identify statistically significant findings that would have been otherwise missed in a smaller sample. To our knowledge this is the first population-based study that assesses the impact of patients, surgical, and facility characteristics on discharge disposition after RC for BCa. This is the first study with this size that assesses factors associated with readmission-providing opportunity to mitigate this in both care provider and administrative level. Additionally, the use of a multivariate analysis allows us to control for several variables that may have otherwise been confounding factors.

## 5. Conclusions

Several specific patient, surgical, and facility characteristics were identified that may significantly impact discharge disposition after RC for bladder cancer. This new information should help guide surgeons and patients with preoperative counseling and shared decision-making process. Prompt identification of patients at risk for non-home discharge can be useful for implementing comprehensive discharge planning protocols that may help with more appropriate and efficient resource allocation.

## Figures and Tables

**Table 1:** Patients and Surgical Characteristics.

	Home Discharge		CFRs		*p* Value
**N. of Patients**	**113,229**	**(82.0%)**	**24,922**	**(18.0%)**	
**Age, years, n (%)**					<0.0001
younger than 55	12,711	(93.5%)	884	(6.5%)	
55–64	27,594	(91.6%)	2545	(8.4%)	
65–69	21,150	(87.0%)	3161	(13.0%)	
70–74	21,854	(81.0%)	5111	(19.0%)	
75 or Older	29,920	(69.4%)	13,221	(30.6%)	
**Gender, n (%)**					<0.0001
Male	94,250	(83.7%)	18,406	(16.3%)	
Female	18,976	(74.4%)	6516	(25.6%)	
**Comorbidity index, n (%)**					<0.0001
CCI = 0	9371	(89.5%)	1105	(10.5%)	
CCI = 1	5160	(84.6%)	937	(15.4%)	
CCI 2 or greater	98,698	(81.2%)	22,880	(18.8%)	
**Race and Etnicity, n (%)**					<0.0001
N-H-White	88,224	(81.6%)	19,836	(18.4%)	
N-H-Black	5780	(81.2%)	1339	(18.8%)	
Hispanic	3089	(82.6%)	651	(17.4%)	
Other	15,902	(84.0%)	3028	(16.0%)	
Unknown	234	(77.5%)	68	(22.5%)	
**Primary insurance, n (%)**					<0.0001
Self-Pay	1958	(95.9%)	83	(4.1%)	
Medicaid	5658	(86.3%)	902	(13.8%)	
Medicare	68,890	(76.7%)	20,907	(23.3%)	
HMO/PPO	32,706	(93.1%)	2424	(6.9%)	
Others	4017	(86.9%)	606	(13.1%)	
**Marital Status**					<0.0001
Married, n (%)	69,539	(86.4%)	10,962	(13.6%)	
Single/Widowed, n (%)	32,701	(73.9%)	11,520	(26.1%)	
Others	10,989	(81.8%)	2440	(18.2%)	
**Surgical Approach**					<0.0001
Robotic, n (%)	22,355	(83.3%)	4477	(16.7%)	
Open, n (%)	90,874	(81.6%)	20,445	(18.4%)	
**Type of Urinary Diversion**					<0.0001
Incontinent n (%)	101,007	(81.4%)	23,113	(18.6%)	
Continent n (%)	6905	(90.9%)	695	(9.1%)	
**LOS, days, mean (SD)/ median (IQR)**	9.7 (6.3)	8.0 (6.0–11.0)	17.2 (13.9)	13.0 (8.0–21.0)	<0.0001
**LOS ≤ 5days n, (%)**	14,501	(95.2%)	733	(4.8%)	<0.0001
**LOS > 5days n, (%)**	98,728	(80.3%)	24,189	(19.7%)	<0.0001

**Table 2:** Facility Characteristics.

	Home Discharge		CFRs		*p* Value
**Hospital volume facility, beds, n (%)**					<0.0001
≤200	10,344	(78.6%)	2824	(21.4%)	
200–299	14,711	(82.4%)	3132	(17.6%)	
300–399	19,171	(82.2%)	4152	(17.8%)	
≥400	69,003	(82.3%)	14,814	(17.7%)	
**Hospital teaching status n (%)**					<0.0001
Teaching	63,303	(82.3%)	13,572	(17.7%)	
Non-teaching	49,926	(81.5%)	11,350	(18.5%)	
**Hospital Location n (%)**					0.1078
Urban	104,272	(81.9%)	23,026	(18.1%)	
Rural	8957	(82.5%)	1896	(17.5%)	
Not reported					
**Annual Hospital Volume n (%)**					<0.0001
High	23,230	(82.4%)	4951	(17.6%)	
Intermediate	67,882	(82.7%)	14,204	(17.3%)	
Low	22,117	(79.3%)	5767	(20.7%)	
**Annual Surgeon Volume n (%)**					<0.0001
High	22,354	(83.9%)	4285	(16.1%)	
Intermediate	69,758	(83.0%)	14,244	(17.0%)	
Low	21,117	(76.8%)	6393	(23.2%)	
**Year of Surgery n (%)**					<0.0001
<2005	32,569	(84.0%)	6187	(16.0%)	
2006	5692	(83.1%)	1161	(16.9%)	
2007	5920	(85.7%)	991	(14.3%)	
2008	6044	(85.0%)	1063	(15.0%)	
2009	6023	(82.7%)	1261	(17.3%)	
2010	5745	(81.1%)	1342	(18.9%)	
2011	5481	(81.9%)	1215	(18.1%)	
2012	5118	(78.9%)	1368	(21.1%)	
2013	5139	(80.1%)	1275	(19.9%)	
2014	5482	(78.5%)	1500	(21.5%)	
2015	5991	(79.3%)	1562	(20.7%)	
2016	6657	(79.0%)	1772	(21.0%)	
2017	6324	(78.5%)	1731	(21.5%)	
2018	6226	(81.2%)	1437	(18.8%)	
2019	4818	(82.0%)	1057	(18.0%)	
**Region n (%)**					<0.0001
Midwest	25,572	(79.0%)	6801	(21.0%)	
Northeast	21,484	(78.6%)	5833	(21.4%)	
South	42,969	(84.5%)	7862	(15.5%)	
West	23,204	(84.0%)	4426	(16.0%)	

## Data Availability

Restrictions apply to the availability of these data. Data were obtained from Premier, Inc., and are available from the authors with the permission of Premier, Inc.

## Appendix A

**Table A1:** Discharge Disposition Definition accordingly to Premiere Healthcare data.

Discharge home with/without home healthcare	DISC HOME W/HOME HEALTH PLAN ACUT IP RDM
	DISCH HOME/SELF PLANNED ACUTE IP READM
	DISCHARGED TO HOME HEALTH ORG.
	DISCHARGED TO HOME IV PROVIDER
	DISCHARGED TO HOME OR SELF CARE
Continue Rehabilitation Centers (CFRs)	DIS/TRAN FACLTY UNLISTD PLAN ACUT IP RDM
	DIS/TRAN MEDC SWING BED PLAN ACUT IP RDM
	DIS/TRAN NURSNG MEDCAID PLAN ACUT IP RDM
	DIS/TRAN PSYCH HOS/DPU PLAN ACUTE IP RDM
	DISC/TRAN CUST/SUPP FAC PLAN ACUT IP RDM
	DISC/TRAN DESIG DISASTR PLAN ACUT IP RDM
	DISC/TRAN SHRT TERM HOS PLAN ACUT IP RDM
	DISC/TRAN SNF MEDICARE PLAN ACUT IP RDM
	DISC/TRANS CANCER/CHILD PLAN ACUT IP RDM
	DISC/TRANS FEDERAL FAC PLAN ACUTE IP RDM
	DISC/TRANS IRF/REH DPU PLAN ACUTE IP RDM
	DISC/TRANS MEDICR LTCH PLAN ACUTE IP RDM
	DISCH/TRANS CAH PLAN ACUTE IP READM
	DISCHARGED TO HOSPICE-HOME
	DISCHARGED TO HOSPICE-MEDICAL FACILITY
	DISCHARGED/TRANSFERRED TO A CAH
	DISCHARGED/TRANSFERRED TO FEDERAL HOSP
	DISCHARGED/TRANSFERRED TO ICF
	DISCHARGED/TRANSFERRED TO OTHER FACILITY
	DISCHARGED/TRANSFERRED TO PSYCH HOSP
	DISCHARGED/TRANSFERRED TO SNF
	DISCHGRD TO THIS INSTITUTION FOR OP SVCS
	DISCHRGD/TRANSFRD TO SWING BED
	DISCHRGD/TRNSFRD TO A NURSING FACILITY M
	DSCHRD/XFERED CANCER CTR/CHILDRN HOSP
	DSCHRD/XFERED OTH HLTH INST NOT IN LIST
	DSCHRGD TO OTHER INSTITUTION FOR OP SVCS
	DSCHRGD/TRNSFRD TO A LTC HOSPITAL
	DSCHRGD/TRNSFRD TO ANOTHER REHAB FACILTY

## Appendix B

**Table A2:** International Classification of Diseases, 9th and 10th (ICD-9 and ICD-10) for procedure codes.

ICD VERSION	ICD CODE	ICD DESCRIPTION
9	17.44	ENDOSCOPIC ROBOTIC ASSISTED PROC
9	56.51	FORM CUTANEOUS URETERO-ILEOSTOMY
9	56.61	FORM CUTANEOUS URETEROSTOMY NEC
9	45.00	INTESTINAL INCISION NOS
9	45.50	INTESTINAL SEGMENT ISOLATION NOS
9	17.42	LAP ROBOTIC ASSISTED PROCEDURE
9	54.21	LAPAROSCOPY
9	56.95	LIGATION OF URETER
9	17.41	OPEN ROBOTIC ASSISTED PROCEDURE
9	56.34	OPEN URETERAL BIOPSY
9	45.71	OPEN/OTHR MULT SEGMT LG BOWEL RESEC
9	17.49	OTHR/UNSPEC ROBOTIC ASSISTED PROC
9	45.62	PARTIAL RESECTION SMALL BOWEL NEC
9	46.23	PERMANENT ILEOSTOMY NEC
9	57.71	RADICAL CYSTECTOMY
9	40.53	RADICAL EXCISE ILIAC LYMPH NODES
9	40.59	RADICAL LYMPH NODE EXCISION NEC
9	40.50	RADICAL LYMPH NODE EXCISION NOS
9	60.5	RADICAL PROSTATECTOMY
9	68.7	RADICAL VAGINAL HYSTERECTOMY
9	68.79	RADICAL VAGINAL HYSTERECTOMY NOS
9	71.5	RADICAL VULVECTOMY
9	66.51	REMOVE BOTH FALLOPIAN TUBES
9	45.91	SM-TO-SM INTESTINAL ANASTOMOSIS
9	46.81	SMALL INTESTINE MANIPULATION
9	45.51	SMALL INTESTINE SEGMENT ISOLATION
9	68.4	TOTAL ABDOMINAL HYSTERECTOMY
9	68.49	TOTAL ABDOMINAL HYSTERECTOMY NOS
9	57.79	TOTAL CYSTECTOMY NEC
9	56.99	URETERAL OPERATIONS NEC
9	56.40	URETERECTOMY NOS
9	56.74	URETERONEOCYSTOSTOMY
9	57.87	URINARY BLADDER RECONSTRUCTION
9	56.71	URINARY DIVERSION TO INTESTINE
10	0T1807B	BP BIL URETERS TO BLADDER W/ATS,OA
10	0T18079	BP BIL URETERS TO COLOCUT W/ATS,OA
10	0T18479	BP BIL URETERS TO COLOCUT W/ATS,PEA
10	0T180Z9	BP BIL URETERS TO COLOCUTANEOUS,OA
10	0T184Z9	BP BIL URETERS TO COLOCUTANEOUS,PEA
10	0T18478	BP BIL URETERS TO COLON W/ATS,PEA
10	0T1807D	BP BIL URETERS TO CUTANE W/ATS,OA
10	0T1847D	BP BIL URETERS TO CUTANE W/ATS,PEA
10	0T180JD	BP BIL URETERS TO CUTANEOUS W/SS,OA
10	0T183JD	BP BIL URETERS TO CUTANEOUS W/SS,PA
10	0T1807C	BP BIL URETERS TO ILEOCUT W/ATS,OA
10	0T1847C	BP BIL URETERS TO ILEOCUT W/ATS,PEA
10	0T184JC	BP BIL URETERS TO ILEOCUT W/SS,PEA
10	0T180ZC	BP BIL URETERS TO ILEOCUTANEOUS,OA
10	0T184ZC	BP BIL URETERS TO ILEOCUTANEOUS,PEA
10	0T184JA	BP BIL URETERS TO ILEUM W/SS,PEA
10	0T180JB	BP BILAT URETERS TO BLADDER W/SS,OA
10	0T180J9	BP BILAT URETERS TO COLOCUT W/SS,OA
10	0T180J8	BP BILAT URETERS TO COLON W/SS,OA
10	0T180JC	BP BILAT URETERS TO ILEOCUT W/SS,OA
10	0T1807A	BP BILAT URETERS TO ILEUM W/ATS,OA
10	0T1847A	BP BILAT URETERS TO ILEUM W/ATS,PEA
10	0T180JA	BP BILAT URETERS TO ILEUM W/SS,OA
10	0T18078	BP BILTRL URETERS TO COLON W/ATS,OA
10	0T1B079	BP BLADDER TO COLOCUTANE W/ATS,OA
10	0T1B479	BP BLADDER TO COLOCUTANE W/ATS,PEA
10	0T1B0Z9	BP BLADDER TO COLOCUTANEOUS,OA
10	0T1B4Z9	BP BLADDER TO COLOCUTANEOUS,PEA
10	0T1B07D	BP BLADDER TO CUTANEOUS W/ATS,OA
10	0T1B07C	BP BLADDER TO ILEOCUTANE W/ATS,OA
10	0T1B47C	BP BLADDER TO ILEOCUTANE W/ATS,PEA
10	0T1B0KC	BP BLADDER TO ILEOCUTANE W/NATS,OA
10	0T1B0JC	BP BLADDER TO ILEOCUTANE W/SS,OA
10	0T1B4JC	BP BLADDER TO ILEOCUTANE W/SS,PEA
10	0T1B0ZC	BP BLADDER TO ILEOCUTANEOUS,OA
10	0T1B4ZC	BP BLADDER TO ILEOCUTANEOUS,PEA
10	0T170ZD	BP LEFT URETER TO CUTANEOUS,OA
10	0T170ZC	BP LEFT URETER TO ILEOCUTANEOUS,OA
10	0T170Z9	BP LT URETER TO COLOCUTANEOUS,OA
10	0T174Z9	BP LT URETER TO COLOCUTANEOUS,PEA
10	0T1707D	BP LT URETER TO CUTANEOUS W/ATS,OA
10	0T170JD	BP LT URETER TO CUTANEOUS W/SS,OA
10	0T1747C	BP LT URETER TO ILEOCUT W/ATS,PEA
10	0T1707C	BP LT URETER TO ILEOCUTANE W/ATS,OA
10	0T170JC	BP LT URETER TO ILEOCUTANE W/SS,OA
10	0T174ZC	BP LT URETER TO ILEOCUTANEOUS,PEA
10	0T1707A	BP LT URETER TO ILEUM W/ATS,OA
10	0T1747A	BP LT URETER TO ILEUM W/ATS,PEA
10	0T170JA	BP LT URETER TO ILEUM W/SS,OA
10	0T16079	BP RT URETER TO COLOCUTANE W/ATS,OA
10	0T160Z9	BP RT URETER TO COLOCUTANEOUS,OA
10	0T164Z9	BP RT URETER TO COLOCUTANEOUS,PEA
10	0T164Z8	BP RT URETER TO COLON,PEA
10	0T1607D	BP RT URETER TO CUTANEOUS W/ATS,OA
10	0T164JD	BP RT URETER TO CUTANEOUS W/SS,PEA
10	0T164ZD	BP RT URETER TO CUTANEOUS,PEA
10	0T1647C	BP RT URETER TO ILEOCUT W/ATS,PEA
10	0T1607C	BP RT URETER TO ILEOCUTANE W/ATS,OA
10	0T160JC	BP RT URETER TO ILEOCUTANE W/SS,OA
10	0T164JC	BP RT URETER TO ILEOCUTANE W/SS,PEA
10	0T160ZC	BP RT URETER TO ILEOCUTANEOUS,OA
10	0T164ZC	BP RT URETER TO ILEOCUTANEOUS,PEA
10	0T1607A	BP RT URETER TO ILEUM W/ATS,OA
10	0T164ZA	BP RT URETER TO ILEUM,PEA
10	0T180ZD	BYPASS BIL URETERS TO CUTANEOUS,OA
10	0T184ZD	BYPASS BIL URETERS TO CUTANEOUS,PEA
10	0T180ZB	BYPASS BILAT URETERS TO BLADDER,OA
10	0T184ZB	BYPASS BILAT URETERS TO BLADDER,PEA
10	0T180Z8	BYPASS BILAT URETERS TO COLON,OA
10	0T184Z8	BYPASS BILAT URETERS TO COLON,PEA
10	0T180ZA	BYPASS BILAT URETERS TO ILEUM,OA
10	0T184ZA	BYPASS BILAT URETERS TO ILEUM,PEA
10	0T1B4ZD	BYPASS BLADDER TO CUTANEOUS,PEA
10	0T170ZB	BYPASS LEFT URETER TO BLADDER,OA
10	0T170ZA	BYPASS LEFT URETER TO ILEUM,OA
10	0T170Z8	BYPASS LT URETER TO COLON,OA
10	0T174Z8	BYPASS LT URETER TO COLON,PEA
10	0T174ZD	BYPASS LT URETER TO CUTANEOUS,PEA
10	0T174ZA	BYPASS LT URETER TO ILEUM,PEA
10	0T160ZB	BYPASS RT URETER TO BLADDER,OA
10	0T160J8	BYPASS RT URETER TO COLON W/SS,OA
10	0T160Z8	BYPASS RT URETER TO COLON,OA
10	0T160ZD	BYPASS RT URETER TO CUTANEOUS,OA
10	0T160ZA	BYPASS RT URETER TO ILEUM,OA
10	0TB70ZZ	EXCISION LT URETER,OPEN APPROACH
10	0TB73ZX	EXCISION LT URETER,PA,DIAGNOSTIC
10	0TB74ZX	EXCISION LT URETER,PEA,DIAGNOSTIC
10	0TB78ZZ	EXCISION LT URETER,VN OR AOE
10	0TB78ZX	EXCISION LT URETER,VN OR AOE,DIAG
10	0TBB0ZX	EXCISION OF BLADDER,OA,DIAGNOSTIC
10	0TBB0ZZ	EXCISION OF BLADDER,OPEN APPROACH
10	0TBB3ZX	EXCISION OF BLADDER,PA,DIAGNOSTIC
10	0TBB4ZX	EXCISION OF BLADDER,PEA,DIAGNOSTIC
10	0TBB7ZZ	EXCISION OF BLADDER,VN OR AO
10	0TBB7ZX	EXCISION OF BLADDER,VN OR AO,DIAG
10	0TBB8ZZ	EXCISION OF BLADDER,VN OR AOE
10	0TBB8ZX	EXCISION OF BLADDER,VN OR AOE,DIAG
10	0DBB8ZZ	EXCISION OF ILEUM NAT/AOE
10	0DBB0ZZ	EXCISION OF ILEUM, OPEN APPROACH
10	0TB70ZX	EXCISION OF LT URETER,OA,DIAGNOSTIC
10	0TB74ZZ	EXCISION OF LT URETER,PERC ENDO APP
10	0TB77ZX	EXCISION OF LT URETER,VN OR AO,DIAG
10	07BC0ZZ	EXCISION OF PELVIS LYMPHATIC,OA
10	07BC3ZZ	EXCISION OF PELVIS LYMPHATIC,PA
10	07BC4ZZ	EXCISION OF PELVIS LYMPHATIC,PEA
10	0TB68ZZ	EXCISION OF RT URETER,VN OR AOE
10	0TBD0ZX	EXCISION OF URETHRA,OA,DIAGNOSTIC
10	0TBD0ZZ	EXCISION OF URETHRA,OPEN APPROACH
10	0TBD3ZX	EXCISION OF URETHRA,PA,DIAGNOSTIC
10	0TBD4ZX	EXCISION OF URETHRA,PEA,DIAGNOSTIC
10	0TBD4ZZ	EXCISION OF URETHRA,PERC ENDO APP
10	0TBD7ZZ	EXCISION OF URETHRA,VN OR AO
10	0TBD8ZZ	EXCISION OF URETHRA,VN OR AOE
10	0TBD8ZX	EXCISION OF URETHRA,VN OR AOE,DIAG
10	0TB60ZX	EXCISION RT URETER,OA,DIAGNOSTIC
10	0TB60ZZ	EXCISION RT URETER,OPEN APPROACH
10	0TB63ZX	EXCISION RT URETER,PA,DIAGNOSTIC
10	0TB64ZX	EXCISION RT URETER,PEA,DIAGNOSTIC
10	0TB64ZZ	EXCISION RT URETER,PERC ENDO APP
10	0TB67ZX	EXCISION RT URETER,VN OR AO,DIAG
10	0TB68ZX	EXCISION RT URETER,VN OR AOE,DIAG
10	0DB80ZZ	EXCISION SMALL INTESTINE OPEN APPRO
10	0DB84ZZ	EXCISION SMALL INTESTINE PEA
10	0DB83ZZ	EXCISION SMALL INTESTINE PERCU APPR
10	0TCB0ZZ	EXTIRPATION OF MATTER BLADDER,OA
10	0TCB4ZZ	EXTIRPATION OF MATTER BLADDER,PEA
10	0TNB0ZZ	RELEASE BLADDER,OPEN APPROACH
10	0TNB4ZZ	RELEASE BLADDER,PERC ENDO APP
10	0DNB0ZZ	RELEASE ILEUM, OPEN APPROACH
10	0DNB4ZZ	RELEASE ILEUM,PERCU ENDO APPR
10	0DN84ZZ	RELEASE SMALL INTESTINE, PEA
10	0DN80ZZ	RELEASE SMALL INTESTINE,OPEN APPR
10	0TND4ZZ	RELEASE URETHRA,PERC ENDO APP
10	0TND7ZZ	RELEASE URETHRA,VN OR AO
10	0TND8ZZ	RELEASE URETHRA,VN OR AOE
10	0TRB07Z	REPLACEMENT OF BLADDER W/ATS,OA
10	0TRB47Z	REPLACEMENT OF BLADDER W/ATS,PEA
10	0TRB0KZ	REPLACEMENT OF BLADDER W/NATS,OA
10	0TRB4KZ	REPLACEMENT OF BLADDER W/NATS,PEA
10	0TRB0JZ	REPLACEMENT OF BLADDER W/SS,OA
10	0TS80ZZ	REPOSITION BILATERAL URETERS,OA
10	0TSC0ZZ	REPOSITION BLADDER NECK,OA
10	0TSC4ZZ	REPOSITION BLADDER NECK,PEA
10	0TSB0ZZ	REPOSITION BLADDER,OPEN APPROACH
10	0TS70ZZ	REPOSITION LT URETER,OPEN APPROACH
10	0TS74ZZ	REPOSITION LT URETER,PERC ENDO APP
10	0TS60ZZ	REPOSITION RT URETER,OPEN APPROACH
10	0DS80ZZ	REPOSITION SMALL INTESTINE,OA
10	0TSD0ZZ	REPOSITION URETHRA,OPEN APPROACH
10	0TT70ZZ	RESECTION LT URETER,OPEN APPROACH
10	0TTC0ZZ	RESECTION OF BLADDER NECK,OA
10	0TTC4ZZ	RESECTION OF BLADDER NECK,PEA
10	0TTC8ZZ	RESECTION OF BLADDER NECK,VN OR AOE
10	0TTB0ZZ	RESECTION OF BLADDER,OPEN APPROACH
10	0TTB4ZZ	RESECTION OF BLADDER,PERC ENDO APP
10	0TTB7ZZ	RESECTION OF BLADDER,VN OR AO
10	0TTB8ZZ	RESECTION OF BLADDER,VN OR AOE
10	0TT74ZZ	RESECTION OF LT URETER,PEA
10	0TT78ZZ	RESECTION OF LT URETER,VN OR AOE
10	0TT64ZZ	RESECTION OF RT URETER,PEA
10	0TT68ZZ	RESECTION OF RT URETER,VN OR AOE
10	0DT80ZZ	RESECTION OF SMALL INTESTINE,OA
10	0DT84ZZ	RESECTION OF SMALL INTESTINE,PEA
10	0TTD0ZZ	RESECTION OF URETHRA,OPEN APPROACH
10	0TTD4ZZ	RESECTION OF URETHRA,PERC ENDO APP
10	0TTD7ZZ	RESECTION OF URETHRA,VN OR AO
10	0TTD8ZZ	RESECTION OF URETHRA,VN OR AOE
10	0UT90ZZ	RESECTION OF UTERUS,OPEN APPROACH
10	0UT94ZZ	RESECTION OF UTERUS,PERC ENDO APP
10	0UT97ZZ	RESECTION OF UTERUS,VN/AO
10	0UTG0ZZ	RESECTION OF VAGINA,OPEN APPROACH
10	0UTG4ZZ	RESECTION OF VAGINA,PERC ENDO APP
10	0UTG7ZZ	RESECTION OF VAGINA,VN/AO
10	0UTG8ZZ	RESECTION OF VAGINA,VN/AOE
10	0VT04ZZ	RESECTION PROSTATE, PERCU ENDO APPR
10	0VT07ZZ	RESECTION PROSTATE, VN/AO
10	0UT00ZZ	RESECTION RT OVARY,OPEN APPROACH
10	0UT04ZZ	RESECTION RT OVARY,PERC ENDO APP
10	0TT60ZZ	RESECTION RT URETER,OPEN APPROACH
10	8E0W3CZ	ROBOTIC ASSISTED PX TRUNK REGION,PA

## Appendix C

**Table A3:** Predictors of discharge disposition to CFRs. Hospital Volume Analysis.

	High Volume Hospitals				Non-High-Volume Hospitals			
	OR	Low 95% CI	High 95% CI	*p*-Value	OR	Low 95% CI	High 95% CI	*p*-Value
**Age, years**				<0.0001				<0.0001
Continue	1.056	1.051	1.061	<0.0001	1.068	1.066	1.071	<0.0001
**Marital Status**				<0.0001				<0.0001
Married, n (%)	ref				ref			
Single/Widowed, n (%)	2.419	2.242	2.61	<0.0001	2.216	2.136	2.229	<0.0001
Others, n (%)	0.843	0.734	0.968	<0.0001	1.738	1.63	1.852	<0.0001
**Gender**				<0.0001				<0.0001
Male	ref				ref			
Female	1.564	1.439	1.7	<0.0001	1.407	1.351	1.466	<0.0001
**Comorbidity index**				<0.0001				<0.0001
CCI = 0	ref				ref			
CCI = 1	1.673	1.378	2.032	0.3538	1.227	1.091	1.308	0.0001
CCI = 2 or greater	2.411	2.083	2.791	<0.0001	2.217	2.042	2.407	<0.0001
**Race, and Etnicity n (%)**				<0.0001				
N-H-White	ref				ref			
N-H-Black	1.177	1.016	1.364	0.9397	1.006	0.929	1.089	0.8775
Hispanic	2.191	1.777	2.702	0.9208	0.885	0.795	0.985	0.0117
Other	1.481	1.309	1.676	0.9327	0.852	0.805	0.901	<0.0001
Unknown	<0.001	<0.001	>999.999	0.9345	1.408	1.04	1.905	0.008
**Primary insurance**				<0.0001				<0.0001
Self Pay	0.288	0.134	0.621	<0.0001	0.819	0.644	1.042	<0.0001
Medicaid	2.195	1.839	2.619	<0.0001	1.906	1.721	2.11	<0.0001
Medicare	1.88	1.674	2.111	<0.0001	1.858	1.751	1.97	<0.0001
HMO/PPO	ref				ref			
Others	2.665	2.066	3.439	<0.0001	1.672	1.495	1.87	<0.0001
**Urinary diversion**				0.4696				<0.0001
Incontinent	ref				ref			
Continent	0.945	0.809	1.102	0.4696	0.741	0.67	0.82	<0.0001
**Hospital size**				<0.0001				<0.0001
≤200	ref				ref			
200–299	1.121	0.739	1.7	0.4429	0.728	0.683	0.776	<0.0001
300–399	1.183	0.89	1.573	0.5197	0.803	0.755	0.854	0.1225
≥400	1.777	1.374	2.297	<0.0001	0.793	0.749	0.838	0.0068
**Hospital teaching status**				<0.0001				<0.0001
Teaching	ref				ref			
Non-teaching	1.523	1.292	1.794	<0.0001	1.209	1.161	1.258	<0.0001
**Hospital Location**				<0.0001				0.0007
Urban	ref				ref			
Rural	0.252	0.189	0.337	<0.0001	0.901	0.849	0.957	0.0007
**Region n (%)**				<0.0001				<0.0001
Midwest	ref			<0.0001	ref			
Northeast	1.12	1.008	1.244	<0.0001	1.343	1.269	1.422	<0.0001
South	0.608	0.54	0.685	0.0008	0.721	0.69	0.753	<0.0001
West	0.361	0.298	0.438	<0.0001	0.746	0.709	0.785	<0.0001
**Surgical approach**				<0.0001				<0.0001
open	ref				ref			
robotic	0.634	0.58	0.693	<0.0001	0.73	0.693	0.768	<0.0001
**Year of Surgery**								
Continue	1.071	1.061	1.081	<0.0001	1.051	1.047	1.055	<0.0001
